# Characterization of the plasma proteome of nonhuman primates during Ebola virus disease or melioidosis: a host response comparison

**DOI:** 10.1186/s12014-019-9227-3

**Published:** 2019-02-07

**Authors:** Michael D. Ward, Ernst E. Brueggemann, Tara Kenny, Raven E. Reitstetter, Christopher R. Mahone, Sylvia Trevino, Kelly Wetzel, Ginger C. Donnelly, Cary Retterer, Robert B. Norgren, Rekha G. Panchal, Travis K. Warren, Sina Bavari, Lisa H. Cazares

**Affiliations:** 1 Molecular and Translational Sciences Division, U.S. Army Medical Research Institute of Infectious Diseases, Frederick, MD 21702 USA; 2 Bacteriology Division, U.S. Army Medical Research Institute of Infectious Diseases, Frederick, MD 21702 USA; 3 Department of Genetics, Cell Biology and Anatomy, University of Nebraska Medical Center, Omaha, NE 68198 USA

**Keywords:** Ebola virus, *Burkholderia pseudomallei*, Quantitative plasma proteomics

## Abstract

**Background:**

In-depth examination of the plasma proteomic response to infection with a wide variety of pathogens can assist in the development of new diagnostic paradigms, while providing insight into the interdependent pathogenic processes which encompass a host’s immunological and physiological responses. Ebola virus (EBOV) causes a highly lethal infection termed Ebola virus disease (EVD) in primates and humans. The Gram negative non-spore forming bacillus *Burkholderia pseudomallei* (*Bp*) causes melioidosis in primates and humans, characterized by severe pneumonia with high mortality. We sought to examine the host response to infection with these two bio-threat pathogens using established animal models to provide information on the feasibility of pre-symptomatic diagnosis, since the induction of host molecular signaling networks can occur before clinical presentation and pathogen detection.

**Methods:**

Herein we report the quantitative proteomic analysis of plasma collected at various times of disease progression from 10 EBOV-infected and 5 *Bp*-infected nonhuman primates (NHP). Our strategy employed high resolution LC–MS/MS and a peptide-tagging approach for relative protein quantitation. In each infection type, for all proteins with > 1.3 fold abundance change at any post-infection time point, a direct comparison was made with levels obtained from plasma collected daily from 5 naïve rhesus macaques, to determine the fold changes that were significant, and establish the natural variability of abundance for endogenous plasma proteins.

**Results:**

A total of 41 plasma proteins displayed significant alterations in abundance during EBOV infection, and 28 proteins had altered levels during *Bp* infection, when compared to naïve NHPs. Many major acute phase proteins quantitated displayed similar fold-changes between the two infection types but exhibited different temporal dynamics. Proteins related to the clotting cascade, immune signaling and complement system exhibited significant differential abundance during infection with EBOV or *Bp*, indicating a specificity of the response.

**Conclusions:**

These results advance our understanding of the global plasma proteomic response to EBOV and *Bp* infection in relevant primate models for human disease and provide insight into potential innate immune response differences between viral and bacterial infections.

**Electronic supplementary material:**

The online version of this article (10.1186/s12014-019-9227-3) contains supplementary material, which is available to authorized users.

## Background

Ebola virus (EBOV) causes a highly virulent systemic disease, Ebola virus disease (EVD) that results in hemorrhagic fever (in both primates and humans) with high fatality rates. Outbreaks of EVD occur primarily in Sub-Saharan Africa, and EBOV (formerly designated as Zaire ebolavirus) is responsible for the largest number of outbreaks of the five known members of the Ebolavirus genus, including the first documented outbreak which occurred in 1976, and the largest outbreak which started in 2013 and was finally contained in 2015 (est. 28,000 cases) [1, 2]. In humans, the general symptomatic profile of EVD resembles that of malaria, yellow fever, Lassa fever and typhoid fever, which are also endemic to Sub-Saharan Africa, thus complicating diagnosis and containment efforts [3]. Although PCR-based blood diagnostic methods have been improved to detect the presence of EBOV RNA, most PCR-based assays do not reach reliable detection levels until 72 h after infection, depending on viral load [4], and a negative PCR in the first 3 days of illness onset does not exclude EBOV infection.

NHP models of EBOV infection have provided the most informative data related to the pathology and host response. In rhesus (*Macaca mulatta*) and cynomolgus (*Macaca fascicularis*) macaques infected with 1000 PFU of EBOV (intramuscular route), viremia is initially detected 3–4 days after infection, often coinciding with a febrile response [5, 6]. Monocytes/macrophages and dendritic cells are the first cell types that are infected; virus then spreads to the regional lymph nodes, liver and spleen by the movement of infected cells and free virus into the bloodstream. Lymphopenia, thrombocytopenia, neutrophilia and coagulopathy develop as EVD progresses [7, 8] along with a pro-inflammatory cytokine/chemokine response [9].

Melioidosis is an illness caused by the soil-dwelling Gram negative non-spore forming bacillus *Burkholderia pseudomallei* (*Bp*) which afflicts both humans and animals [10]. Most cases originate in Southeast Asia and Northern Australia where it is a common cause of pneumonia, likely due to aerosolization during monsoon rainfall [11]. A low infectious dose by the aerosol route with the potential for rapid, severe and frequently fatal pneumonia makes *Bp* a bio-threat that necessitates rapid diagnostic strategies. Melioidosis has varied clinical presentations in both humans and non-human primates, including asymptomatic infection, localized skin ulcers/abscesses, chronic pneumonia, and fulminant septic shock with abscesses in multiple internal organs [12, 13]. Treatment of melioidosis is difficult, due to the fact that *Bp* is naturally resistant to multiple antibiotics and prolonged antibiotic treatment (5–6 months) is necessary to prevent relapse.

Although there is no universally accepted NHP model for melioidosis, upon aerosol exposure with *Bp*, rhesus macaques develop progressive pneumonia and sepsis similar to the disease course in humans [14, 15]. The infection can be lethal in rhesus macaques, but like humans, NHPs vary greatly in their response to *Bp* infection and many develop sub-acute pneumonia. *Bp* is an intracellular pathogen that can multiply within phagocytes, including neutrophils, monocytes and macrophages without activating a bactericidal response [16, 17]. Localized disease, such as pneumonia and abscesses are typical in both human cases and the NHP model; however, *Bp* can spread to secondary sites, including liver, spleen and brain, or to the blood, and often results in chronic persistent infection [18, 19]. There have been few reports examining the transcriptomic or proteomic response to melioidosis in humans [20–22].

Characterizing the host response to infection theoretically holds promise for pre-symptomatic diagnosis, since the induction of host molecular signaling networks often occurs before clinical presentation and pathogen detection [23]. Specifically, analyzing changes in host gene and protein expression during infection can generate pathogen-specific biomarker profiles, as different infectious agents may elicit distinct responses. The interrogation of the circulatory host response to EBOV or *Bp* infection in humans has been performed on a small number of samples, and is further complicated by supportive care treatments [24–27]. Therefore, the use of comparable NHP models is necessary for the characterization of the plasma proteomic response. Furthermore, in-depth examination of the host response to various pathogenic organisms generates information that extends beyond simple diagnosis, especially in the context of animal model development and therapeutic evaluation. For example, blood-based host response markers of infection (genetic or protein-based) can be used to better define pathogenesis, stratify disease states and define specific trigger-to-treat paradigms for new therapeutic treatments in animal models of infection. Furthermore the examination of the temporal kinetics of the host response during infection provides data related to virulence determination allowing for the down-selection of strains or isolates used as challenge material for animal model studies.

To track and characterize plasma proteomic host response dynamics, we examined serially collected samples from 10 rhesus macaques during EBOV infection and 5 rhesus macaques during *Bp* infection. Our strategy employed high resolution LC–MS/MS and a peptide-tagging approach for relative protein quantitation. These studies provide a detailed characterization of the blood-based host proteomic response profile to EBOV and *Bp* infection in NHP models which approximate EVD and melioidosis in humans, and highlight the differences in the innate immune response to a lethal viral versus a pathogenic bacteria.

## Materials and methods

### Animal use and ethics statement

All NHP studies were conducted under an IACUC-approved protocol in compliance with the Animal Welfare Act, PHS Policy, and other Federal statutes and regulations relating to animals and experiments involving animals. The facility where this research was conducted is accredited by the Association for Assessment and Accreditation of Laboratory Animal Care, International and adheres to principles stated in the Guide for the Care and Use of Laboratory Animals, National Research Council, 2011. Research was conducted under IACUC-approved protocols in compliance with the Animal Welfare Act, PHS Policy, and other Federal statutes and regulations relating to animals and experiments involving animals.

### EBOV infection

Ten adult rhesus macaques (6 male and 4 female, weight 4.7–5.6 kg, average age 4.2 years) were inoculated with a target titer of 1000 plaque-forming units (PFU) of EBOV (H.sapiens-tc/COD/1995/Kikwit-9510621 (15) demonstrated to be primarily the 8U variant at the mRNA editing site) in 0.5 mL by intramuscular (IM) injection in the left or right quadricep. These animals served as control animals in therapeutic studies, and the samples were retrospectively analyzed to characterize the proteomic host response to EBOV infection. In all animals, plasma collection occurred on Day 0 (pre-infection) and Days 2, 3, 4, 5 and 6 post-infection. All EBOV studies were conducted in Animal Biosafety Level 4 containment. Beginning on Day 0 and continuing for the duration of the in-life phase, clinical observations were recorded, and animals were closely monitored for disease progression. Moribund animals were humanely euthanized based on institutional-approved clinical scoring and pre-determined endpoints.

### EBOV RT-PCR

For quantitative assessment of viral RNA, whole blood was collected using a K3EDTA Greiner Vacuette tube (or equivalent) and centrifuged at 2500 (± 200) relative centrifugal force for 10 ± 2 min. To inactivate virus, plasma was treated with 3 parts (300 μl) TriReagent LS and samples were transferred to frozen storage (− 60 °C to − 90 °C), until removal for RNA extraction. Carrier RNA and QuantiFast High Concentration Internal Control (Qiagen) were spiked into the sample before extraction, according to manufacturer’s instructions. Viral RNA was eluted in AVE buffer. Each extracted RNA sample was tested with the QuantiFast Internal Control RT-PCR RNA Assay (Qiagen) to evaluate the yield of the spiked-in QuantiFast High Concentration Internal Control. If the internal control amplified within manufacturer-designated ranges, further quantitative analysis of the viral target was performed. RT–PCR was conducted using an ABI 7500 Fast Dx using primers specific to EBOV glycoprotein. Samples were run in triplicate. For quantitative assessments, the average of the triplicate genomic equivalents (GE) per reaction were determined and multiplied by 800 to obtain GE ml^−1^ plasma. Standard curves were generated using synthetic RNA. The limits of quantification for this assay are 8.0 × 10^4^ − 8.0 × 10^10^ GE ml^−1^ of plasma.

### *Bp* challenge and bacterial load determination

Rhesus macaques (n = 5 adults, 3 Female, 2 Male, weight 5.7–6.4 kg, average age 4.8 years) were exposed using a head-only aerosol exposure system, the NHP were exposed to an average of 346/118 CFU of *B. pseudomallei* HBPUB10134a (dose range: 248–531/71–214 CFU). This closely approximates the target dose of 400 CFU. All animals were exposed on the same day. Exposure durations ranged from 5 to 15 min. The aerosol respiratory deposition fraction was assumed to be 100%. After aerosol exposure, the head of each animal was wiped with a soap solution to remove deposited aerosol, and animals were housed individually under biosafety level 3 conditions. To facilitate collection of blood, central venous catheters (CVC) were placed 15–16 days prior to exposure. Blood was collected for baseline values and once daily for the first 7 days post-exposure and plasma collection for proteomic evaluation occurred on Day 0 (pre-infection) and Days 1, 3, 5, 7, and 9 post-infection. Animals that survived the acute infection were monitored for 46–47 days post-exposure (study endpoint) before euthanization.

### *Bp* bacterial load determination

Two 100 μl samples of whole blood from days 1–7, 14, 21, 28, 35, 42 post-exposure were plated on 5% Sheep blood agar to detect bacteremia. Each plate was incubated at 37 °C for 72 h. Tissue samples from the lung, liver, spleen, pancreas and gonads were collected at necropsy under sterile conditions and cultured for bacteria. Each tissue sample of approximately 0.25–1.0 g was manually homogenized in 2 ml PBS and two 100 μl samples were plated on 5% Sheep blood agar (Thermo-Fisher) to evaluate positive or negative growth.

### Naïve NHP plasma collection

Plasma was collected from five uninfected, healthy naïve rhesus macaques (average age 6 years, average weight 5.3 kg) daily for 9 days to establish a longitudinal set of samples.

### Plasma TMT sample preparation

Plasma samples (6 time points/animal) were first processed in BSL-3 or BSL-4 containment by adding 25 µL SDS-PAGE solubilizing/reducing buffer to 75 µL sample and heating to 95 °C for 10 min. Samples were then removed from containment and stored at − 80 °C until processed by the iFASP method [28]. Briefly, 5 µL of each inactivated plasma sample was added to 200 µL 8 M Urea/100 mM Tris–HCL pH 8.5 (Solution UT8) and filtered through a Microcon-30 kDa Centrifugal Filter Unit with an Ultracel-30 membrane (Millipore, MRCF0R030) at 14,000 × G for 15 min. Following several washing steps with 100 mM Tris pH 8.0, proteins were alkylated with 55 mM Iodoacetamide and digested with 4 µg Trypsin/Lys-C (Promega, V5071) overnight at 37 °C. TMT 6-Plex labeling (Thermo Fisher, 90061) was performed directly on the FASP filters per the manufacturer’s instructions. All 6 single labelled samples were then combined at an equal volume, purified by C18 spin column, dried to completion by speed-vac and stored at − 20 °C until analyzed by LC MS/MS.

### LC–MS/MS TMT analysis

Sample digests were re-suspended in 240 μL of 0.1% formic acid. A Dionex 3000 RSLCnano system (Thermo Scientific) injected 5 μL of each digest onto a pre-column (C18 PepMap 100, 5 μm particle size, 5 mm length × 0.3 mm internal diameter) using a flow rate of 10 µL/min. The loading solvent was 0.1% formic acid in HPLC grade water. Peptides were then loaded onto an Easy-Spray analytical column (15 cm × 75 um) packed with PepMap C18, 3 um particle size, 100 A porosity particles (Thermo Scientific, Inc.). A 2–38% B gradient elution in 160 min was formed using Pump-A (0.1% formic acid) and Pump-B (85% acetonitrile in 0.1% formic acid) at a flow rate of 300 nL/min. The column eluent was connected to an Easy-Spray source (Thermo Scientific) with an electrospray ionization voltage of 2.2 kV. An Orbitrap Elite mass spectrometer (Thermo Scientific, Inc.) with an ion transfer tube temperature of 300 °C and an S-lens setting of 55% was used to focus the peptides. A top 10 data dependent MS/MS method was used to select the 10 most abundant ions in a 400–1600 amu survey scan (120,000 resolution FWHM at m/z 400) with a full AGC target value of 1e6 ions and a maximum injection time of 200 ms. Higher Energy Collisional Dissociation (HCD) MS/MS spectra were acquired at a resolution of 30,000 (FWHM at m/z 400) with an AGC target value of 5e4 ions and a maximum injection time of 200 ms. The isolation width for MS/MS HCD fragmentation was set to 2 Daltons. The normalized HCD Collision energy was 40% with an activation time of 0.1 ms. The dynamic exclusion duration was 30 s.

### Database search and protein quantitation

Acquired MS/MS protein searches were performed with ProteomeDiscoverer 2.1 Sequest HT (Thermo Scientific) using a Human (taxID 9606) and rhesus macaque subset of the SwissProt_2017_01_18 database containing 42,055 sequences, as well as a custom macaque proteome developed at the University of Nebraska Medical Center. Sources for the custom macaque proteome database included the following: MacaM genome (version 7), Zimin et al. [78] and NCBI. Variable modifications used were TMT 6-plex (N-terminal, K), Carbamyl (KMR), Methyl (DE), Acetyl (K), Deamidated (NQ), and Oxidation (M). Cysteine carbamidomethylation was specified as a constant modification. The peptide-level false discovery rate (FDR) was set at 0.1% using Posterior Error Probability validation. Only proteins having at least 2 Peptide Spectral Matches (PSM) were considered, with both unique and razor peptides used for protein quantitation. Normalization by total peptide amount was used with control channel average scaling mode turned on. Mass tolerances were 10 ppm for the MS1 scan and 0.6 Da for all MS/MS scans. Quantitation results were filtered such that only high-confidence/unambiguous PSMs having MS2 isolation interference values equal to or less than 30% were used.

### Western blot analysis

Western blot assays were performed using a mouse monoclonal antibody for galectin-3 binding protein (LGALS3BP) purchased from Origene (Cat# TA503455). Briefly, inactivated plasma samples (2 µl) were run under reducing conditions on a 4–12% precast polyacrylamide gel (NuPAGE bis–tris Thermo-Fisher Cat# NP0321BOX) and transferred to PVDF membranes. Each blot was blocked overnight with blocking buffer in phosphate buffered saline (PBS) (Cat# 37572, Thermo-Fisher) and then incubated with primary antibody against G3BP (1:500) overnight at 4 °C on a rocking platform. After washing 3 × with PBS + 0.1% Tween-20 for 5 min, secondary antibody (1:5000) goat α-mouse IRDye^®^ 680 labelled (LICOR) was added and the blots were incubated an additional hour. The blots were again washed 3 × with PBST, and then stored in PBS until visualized with an Odyssey infrared imaging system (LI-COR Biosciences Lincoln, NE: model number 9210).

### Statistical analysis

Statistical analysis was performed with GraphPad Prism for Windows Version 7.00. All plasma protein abundance data from serially collected plasma samples in response to EBOV or *Bp* were tested for normality using the Shapiro–Wilk test and log-transformed to achieve normal distribution if necessary. An identical test was performed for the protein abundance data from naïve NHP. For all protein abundances with fold changes > 1.3 in at least one post-infection time point in 40% of each cohort, a direct comparison was made to plasma which was sampled on consecutive days from naïve NHPs to determine significance using 2-way ANOVA. A *p* value of < 0.05 was considered significant. To compare the host-response between NHPs exposed to EBOV or *Bp*, protein abundance ratios were compared for overlapping post-infection time points (Days 3, 5 and 6/7 PI) using 2-way ANOVA. An adjustment was performed on all *p*-values for multiple comparisons using the two-stage linear step-up procedure of Benjamini, Krieger and Yekutieli and a false discovery rate (FDR) of 5%. A heat map was generated which displays the average fold change value of proteins for comparison of the NHP host response to EBOV or *Bp*-infection. Each entry was scaled individually to provide a visual representation of the relative abundance of each protein.

## Results

### Sample cohorts

**Fig. 1:**
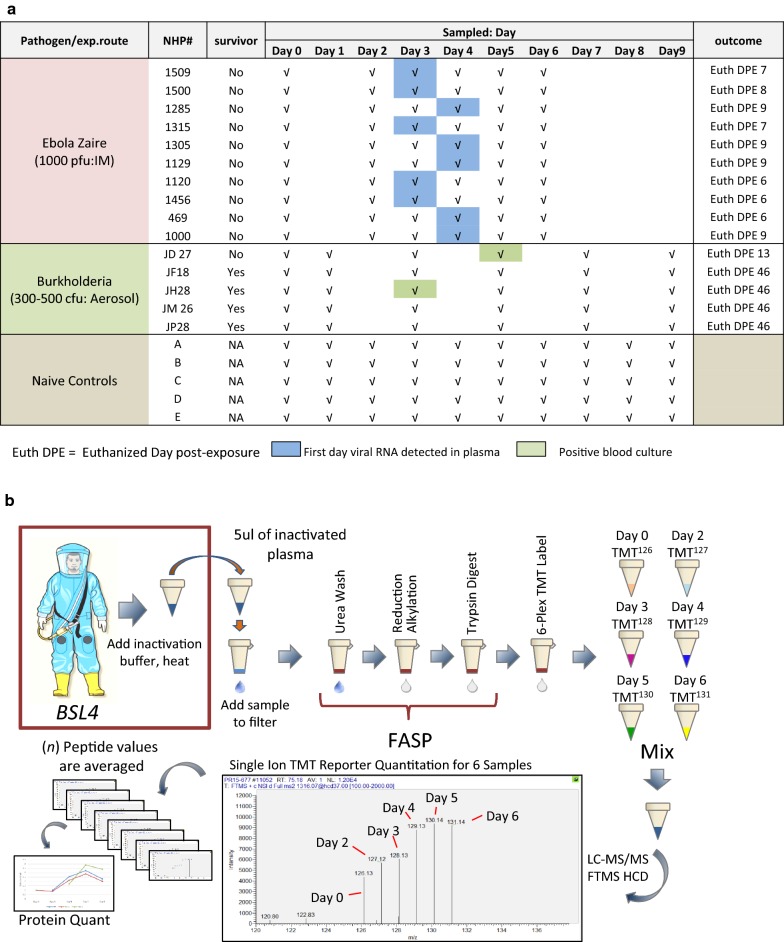
Sample complement and workflow of experimental design to track the NHP proteomic response during infection with EBOV or *Burkholderia pseudomallei*. **a** NHP plasma samples used for this study including designations for the first day of detectable viremia or positive blood culture as well as outcome. **b** Sample processing workflow. SDS PAGE sample buffer and heat were used to inactivate pathogens in plasma samples serially collected from EBOV- or *Bp*-infected rhesus macaques. Filter-assisted sample prep (FASP) was employed to remove the buffer and perform reduction/alkylation, trypsin digestion and TMT labelling. After TMT labelling, serially collected samples from each NHP were mixed together allowing the simultaneous analysis of samples from 6 post-infection time points in a single LC–MS/MS run

As shown in Fig. 1a, the samples for this study encompass 3 independent cohorts of rhesus macaques: EBOV-infected (n = 10), *Bp*-infected (n = 5), and naïve/uninfected (n = 5). Each infected NHP sample set contained a total of 6 serially collected plasma samples: one pre-infection (Day 0) and 5 post-infection (PI) time points. Plasma samples from Naïve NHP were collected daily over a sampling period of 9 days.

Intramuscular infection of rhesus macaques with 1000 PFU of EBOV resulted in all NHPs becoming febrile between Days 3–5 PI and mortality occurred between Days 6 and 9 PI. Necropsy findings and gross pathological changes were consistent with the observations reported previously for EVD in NHP [30]. All non-surviving animals had positive plasma viral RNA values by Days 3 or 4 PI (Fig. 1a and Additional file 1: Table S1). Aerosol infection of rhesus macaques with *Bp* (~ 400 CFU inhaled dose) resulted in symptom onset on Day 3 PI in most animals with acute infection on Days 3–13 PI. Fever (> 1.5 °C above baseline) was present approximately 30% of the time beginning on Day 2 PI in *Bp*-infected NHP. All animals developed significant pulmonary lesions, with 20% mortality (1/5); the remaining 4/5 animals developed chronic infection but survived until the study end-point. The blood culture results for most animals in this study were negative (see Additional file 1: Table S2), with the exception of NHPs JD27 and JH28. By histopathologic examination, the tissues affected most frequently were the lung and associated draining lymph nodes, liver and spleen (see Additional file 1: Table S3).

### Plasma protein quantitation

**Fig. 2:**
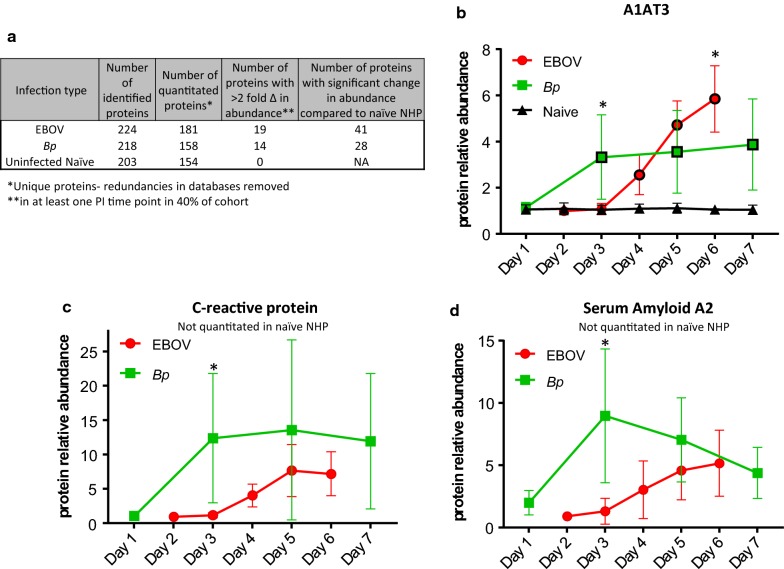
Overview of protein identification/quantitation results and major acute phase protein levels in rhesus macaques infected with EBOV or *Bp*. **a** Number of proteins quantitated, and number of significant protein alterations in EBOV and *Bp*-infected plasma samples when compared to naïve NHP. **b**–**d** The relative protein abundances (y-axis) of **b** alpha-1 anti-trypsin member 3 (A1AT3), **c** C-reactive protein (CRP), and **d** serum amyloid A2 (SAA2) detected in plasma during EBOV or *Bp* infection are plotted versus post-infection day (x-axis). For all three proteins, similar maximum fold change increases were observed between the two infection types but different temporal kinetics contributed to higher levels observed at Day 3 and Day 6 PI for A1AT3, and Day 3 for CRP and SAA2 in the *Bp*-infected NHP. Abundance levels that were significantly different from levels found in naïve NHPs are designated with a black border around the symbol and levels that were significantly different between EBOV- and *Bp*-infected NHPs are designated with an asterisk (*). Statistical significance was based on 2-way ANOVA analysis

To characterize the plasma proteomic response to EVD and melioidosis in rhesus macaques, a semi-quantitative peptide tagging approach was employed to provide the relative abundance of plasma proteins in an analytical workflow using LC–MS/MS. For this study, we chose not to deplete abundant serum proteins due to sample volume constraints. Hence, we expected to identify and quantitate predominately acute phase reactant proteins which are part of the innate immune response. Serially collected EBOV-infected plasma samples were inactivated for removal from Bio-safety Laboratory level 4 (BSL-4) and processed using the strategy depicted in Fig. 1b. The *Bp*- *infected* samples were processed in an identical fashion in BSL-3 containment. On average, a total of 224 ± 36 proteins were identified in EBOV-infected plasma samples (see Fig. 2a), and similar protein total averages (218 ± 41) were obtained for *Bp*-infected plasma samples. Proteins identified in each sample were analyzed for changes in abundance at post-infection time points by using the pre-infection (Day 0) sample as the baseline value (assigned a value of 1.0). The ratios of the intensity of the reporter ions associated with the post-infection time points versus the intensity of the reporter ion in the pre-infection sample were acquired as relative peptide abundance. For all proteins with > 1.3 fold abundance change at any PI time point, a direct comparison was made with abundance ratios from 5 naïve rhesus macaques. This was done to determine the fold changes that were significant in the infected animals, and establish the natural variability of abundance for endogenous plasma proteins using our FASP/TMT strategy. For proteins that were not detected in the Naïve NHP data-set, abundances were considered significantly altered if ratios were 2-fold higher or lower (abundance ratio of 0.5) when compared to the pre-infection sample at any PI time point. To focus on the most commonly altered proteins during infection, we report the results of significant proteins (as compared to naïve NHP levels where applicable or ≥ twofold altered) that were quantitated in at least 40% of each cohort: 4/10 EBOV-infected NHPs and 2/5 *Bp*-infected NHPs. The final number of quantitated proteins in each cohort is shown in Fig. 2a. Proteins with ≥ 2 fold abundance change from pre-infection levels in at least one post-infection time point during EBOV or *Bp* infection in rhesus macaques are shown in Table 1a (EBOV data) and Table 1b (*Bp* data) All proteins with < 2 fold abundance change are listed in Table 2a (EBOV data) and Table 2b (*Bp* data).

**Table 1:** Proteins with > 2 fold abundance alteration with significant abundance change (a) in at least 4/10 EBOV infected NHP when compared to Naive NHP, (b) in at least 2/5 Bp infected

Accession	Description	# NHP quant.	Average CV %	Abundance ratio—Day PI					*p* Value versus naive—Day PI			
				Day 2	Day 3	Day 4	Day 5	Day 6	Day 3	Day 4	Day 5	Day 6
P63261	Actin, cytoplasmic 2	8	5.9	0.97	0.99	1.06	1.63	4.41	Not quantitated in naive NHP plasma			
ORM1_5004	Alpha-1 acid glycoprotein	10	13	1.03	1.27	2.55	3.91	4.82	NS	NS	0.0131	0.0025
SERPINA3_12	Alpha-1 antitrypsin, member 3	10	7.2	0.97	1.03	2.44	4.89	5.76	NS	0.0048	0.0003	0.0003
AHSG_197	Alpha-2-HS-glycoprotein	9	1.1	1.00	0.93	0.74	0.65	0.51	NS	NS	0.0061	0.0003
APOA4_337	Apolipoprotein A-IV	6	2.3	0.95	0.96	0.86	0.52	0.64	NS	NS	0.0058	0.1506
APOA1_335	Apolipoprotein A-I	10	0.58	0.95	0.93	0.81	0.65	0.51	NS	NS	NS	0.0003
P04114	Apolipoprotein B-100	7	0.59	1.10	1.05	0.97	1.24	2.16	NS	NS	0.0022	0.0003
APOE_348	Apolipoprotein E	10	0.70	0.96	0.98	1.10	1.66	2.50	NS	NS	0.0070	0.0003
CP_1356	Clusterin	10	0.62	0.98	0.96	0.96	1.18	1.98	NS	NS	0.0032	0.0003
CLU_1191	Complement C1R subcomponent	9	0.81	0.95	1.09	1.28	2.01	1.67	NS	NS	0.0018	0.0298
CRP_1401	C-reactive protein	8	23	1.02	1.07	3.68	7.55	6.99	Not quantitated in naive NHP plasma			
HP_3240	Haptoglobin	10	4.6	1.07	1.20	1.71	2.31	2.13	NS	0.0484	0.0018	0.0023
HPX_3263	Hemopexin	10	3.1	1.02	1.08	1.40	2.08	2.47	NS	0.0538	0.0003	0.0003
LGALS3BP_3959	Galectin-3 binding protein	7	1.0	1.04	0.84	1.11	1.47	2.57	Not quantitated in naive NHP plasma			
LRG1_116844	Leucine-rich alpha-2-glycoprotein 1	7	13	1.04	1.12	2.20	3.88	3.40	Not quantitated in naive NHP plasma			
LBP_3929	Lipopolysaccharide binding protein	7	19	0.83	0.97	1.45	2.04	2.41	Not quantitated in naive NHP plasma			
P05109	S100 calcium binding protein A8	8	0.74	0.88	1.02	1.19	1.96	4.95	Not quantitated in naive NHP plasma			
S100A9_6280	S100 calcium binding protein A9	4	18	1.00	1.04	1.13	2.57	8.43	Not quantitated in naive NHP plasma			
SAA2_6289	Serum amyloid A2	9	16	0.88	0.91	2.44	4.29	4.50	Not quantitated in naive NHP plasma			

Accession	Description	# NHP quant.	Average CV %	Abundance ratio—Day PI					*p* Value versus naive—Day PI			
				Day 1	Day 3	Day 5	Day 7	Day 9	Day 3	Day 5	Day 7	Day 9
APOA2_336	Apolipoprotein A-II	4	2.7	0.86	0.66	0.51	0.44	0.39	0.0345	NS	0.0131	0.0003
ORM1_5004	Alpha-1 acid glycoprotein	5	30	1.13	2.49	2.96	3.53	3.53	NS	0.0222	0.0030	0.0046
SERPINA3_12	Alpha-1 antitrypsin, member 3	5	30	1.01	2.73	3.01	3.14	3.21	0.0191	0.0121	0.0046	0.0244
P0C0L5	Complement C4-B	5	12	1.72	1.80	1.92	1.98	2.11	Not quantitated in naive NHP plasma			
CRP_1401	C-reactive protein	3	> 100%	0.91	10.98	11.20	10.87	6.99	Not quantitated in naive NHP plasma			
FGA_2243	Fibrinogen alpha chain	5	13	1.13	2.30	2.38	2.27	2.22	0.0018	0.0018	0.0020	0.0056
FGB_2244	Fibrinogen beta chain	5	8.0	1.06	1.85	2.09	1.90	1.95	0.0013	0.0003	0.0003	0.0007
HP_3240	Haptoglobin	5	10	1.02	2.33	2.81	3.13	3.04	0.0189	0.0023	0.0023	0.0043
ITIH1_3697	Inter-alpha-trypsin inhibitor HC 1	4	6.0	0.74	0.60	0.51	0.44	0.40	0.0054	0.0018	0.0005	0.0007
LBP_3929	Lipopolysaccharide binding protein	4	33	1.07	2.12	2.25	2.17	2.01	Not quantitated in naive NHP plasma			
LUM_4060	Lumican	4	6.2	1.30	0.58	0.30	0.31	0.27	NS	0.0284	0.0148	NS
P06702	S100 calcium binding protein A9	2	14	1.14	2.08	2.90	4.36	5.85	Not quantitated in naive NHP plasma			
SAA2-SAA4_100528017	SAA2-SAA4 readthrough	5	7.7	1.55	3.46	2.77	1.99	2.19	Not quantitated in naive NHP plasma			
SAA2_6289	Serum amyloid A2	5	55	1.71	8.56	7.20	5.56	4.98	Not quantitated in naive NHP plasma			

*NS* not significant

**Table 2:** Proteins with < 2 fold alteration with significant abundance change in (a) at least 4/10 EBOV infected NHP when compared to naive NHP, (b) at least 2/5 Bp infected NHP when compared to naive NHP

Accession	Description	# NHP quant.	Average CV %	Abundance ratio—Day PI					*p* Value versus naive—Day PI		
				Day 2	Day 3	Day 4	Day 5	Day 6	Day 4	Day 5	Day 6
AFM_173	Afamin	9	1	1.00	1.02	1.00	0.83	0.79	0.0406	0.0005	0.0002
ALB_213	Albumin	10	0.73	1.01	1.10	0.92	0.76	0.67	0.0441	0.0002	0.0002
SERPINA1_5265	Alpha-1 antitrypsin member 1	10	0.61	0.99	1.03	1.12	1.30	1.37	NS	0.0002	0.0002
APOA2_336	Apolipoprotein A-II	7	2.1	1.09	0.90	0.77	0.59	0.55	0.0065	0.0011	0.0002
APOB_338	Apolipoprotein B48	10	1.8	1.02	0.94	0.94	1.15	1.61	NS	0.0091	0.0002
APOC3_345	Apolipoprotein C-III	6	3	0.99	0.92	0.63	0.67	0.77	0.0132	0.0024	0.0033
APOH_350	Apolipoprotein H	6	1.6	1.03	1.03	0.82	0.60	0.59	0.0020	0.0002	0.0002
SERPING1_710	C1 inhibitor member 1	10	0.34	0.98	0.98	1.06	1.14	1.37	NS	0.0106	0.0002
CPN2_1370	Carboxypeptidase N subunit 2	6	1.3	0.95	1.04	1.01	1.11	1.43	NS	NS	0.0012
CP_1356	Ceruloplasmin (ferroxidase)	10	1	1.00	1.04	1.14	1.29	1.42	NS	0.0436	0.0023
C1S_716	Complement C1S subcomponent	6	1.3	0.97	1.01	1.20	1.48	1.69	NS	NS	0.0133
C2_717	Complement component 2	6	1.1	0.98	0.97	1.01	1.17	1.60	NS	NS	0.0135
C9_735	Complement component 9	10	1.2	1.06	1.02	1.15	1.44	1.58	0.0106	0.0007	0.0004
CFB_629	Complement factor B	10	1.8	1.03	1.07	1.28	1.55	1.90	0.0095	0.0002	0.0002
FGA_2243	Fibrinogen alpha chain	10	0.64	1.00	1.02	1.26	1.38	1.10	0.0413	0.0183	NS
FGB_2244	Fibrinogen beta chain	10	0.5	0.97	1.03	1.22	1.46	1.07	NS	0.0051	NS
FGG_2266	Fibrinogen gamma chain	10	0.62	1.04	1.02	1.22	1.39	1.17	0.0295	0.0031	NS
FN1_2335	Fibronectin 1	10	1.1	0.99	0.93	0.75	0.74	0.78	0.0023	0.0033	0.0095
FETUB_26998	Fetuin B	8	1.8	0.91	0.97	0.83	0.62	0.60	NS	0.0195	0.0320
HRG_3273	Histidine rich glycoprotein	10	0.71	0.97	0.96	0.84	0.71	0.60	0.0514	0.0050	0.0003
ITIH1_3697	Inter-alpha-trypsin inhibitor HC 1	10	0.44	0.98	0.91	0.87	0.80	0.68	0.0022	0.0002	0.0002
ITIH2_3698	Inter-alpha-trypsin inhibitor HC 2	10	0.8	0.98	0.97	0.96	0.73	0.64	0.0307	0.0013	0.0002
ITIH3_3699	Inter-alpha-trypsin inhibitor HC 3	10	0.53	0.99	1.02	1.03	1.18	1.40	NS	0.0062	0.0004
ITIH4_3700	Inter-alpha-trypsin inhibitor HC4	10	1.2	1.00	1.01	1.10	1.21	1.36	NS	NS	0.0037
KNG1_3827	Kininogen 1	6	2	0.98	1.03	0.85	0.63	0.52	NS	0.0078	0.0005
LUM_4060	Lumican	8	1.3	1.02	0.96	0.71	0.60	0.68	NS	0.0418	0.0066
PLG_5340	Plasminogen	10	1	0.95	1.00	0.91	0.74	0.67	NS	0.0008	0.0003
TF_7018	Transferrin	10	0.61	0.99	1.00	0.95	0.82	0.70	0.0389	0.0002	0.0002
TTR_7276	Transthyretin	10	0.6	0.95	0.88	0.82	0.74	0.80	0.0498	0.0128	0.0497
GC_2638	Vitamin D binding protein	10	0.84	1.03	1.03	0.97	0.82	0.77	NS	0.0033	0.0007

Accession	Description	# NHP quant.	Average CV %	Abundance ratio—Day PI					*p* Value versus naive—Day PI			
				Day 1	Day 3	Day 5	Day 7	Day 9	Day 3	Day 5	Day 7	Day 9
AFM_173	Afamin	5	3.5	1.10	0.85	0.82	0.69	0.77	0.0209	0.0051	0.0006	0.0007
ALB_213	Albumin	5	3.3	0.96	0.77	0.63	0.61	0.57	0.0307	0.0025	0.0009	0.0025
SERPINA1_5265	Alpha-1 antitrypsin member 1	5	7.5	1.19	1.49	1.86	1.77	1.89	0.0297	0.0071	0.0025	0.0058
APOA1_335	Apolipoprotein A-I	5	3.2	1.13	0.81	0.59	0.54	0.55	NS	0.0229	0.0101	0.0095
APOH_350	Apolipoprotein H	5	8.2	0.86	0.63	0.62	0.65	0.69	0.0002	0.0002	0.0002	0.0002
CPN2_1370	Carboxypeptidase N subunit 2	3	3.4	1.20	1.33	1.57	1.56	1.59	0.0011	0.0006	0.0002	0.0002
CP_1356	Ceruloplasmin (ferroxidase)	5	4.8	1.05	1.32	1.62	1.59	1.73	0.0354	0.0025	0.0018	0.0023
C3_718	Complement component 3	5	2.9	1.03	1.22	1.29	1.37	1.30	NS	0.0068	0.0008	0.0038
C4A_720	Complement component 4A	5	4.6	1.22	1.31	1.52	1.35	1.22	0.0307	0.0022	0.0094	NS
C5_727	Complement component 5	5	3.0	1.27	1.14	1.36	1.33	1.46	NS	0.0043	0.0068	0.0009
C9_735	Complement component 9	5	5.3	1.06	1.36	1.57	1.62	1.61	NS	0.0033	0.0004	0.0024
CFB_629	Complement factor B	5	4.8	0.97	1.26	1.30	1.35	1.26	0.0155	0.0015	0.0125	0.0498
FGG_2266	Fibrinogen gamma chain	5	3.3	0.90	1.60	1.79	1.88	1.88	0.0005	0.0002	0.0002	0.0010
FN1_2335	Fibronectin 1	5	5.1	0.99	0.71	0.78	0.78	0.78	0.0002	0.0002	0.0012	NS
HPX_3263	Hemopexin	5	7.9	0.96	1.44	1.55	1.79	1.53	0.0118	0.0029	0.0039	0.0420
HRG_3273	Histidine rich glycoprotein	5	5.3	1.01	0.80	0.60	0.56	0.56	0.0418	0.0002	0.0002	0.0002
ITIH2_3698	Inter-alpha-trypsin inhibitor HC 2	4	3.3	0.95	0.71	0.68	0.66	0.62	0.0183	0.0013	0.0004	0.0005
ITIH3_3699	Inter-alpha-trypsin inhibitor HC 3	5	7.3	1.08	1.25	1.51	1.47	1.59	0.0227	0.0093	0.0094	0.0070
ITIH4_3700	Inter-alpha-trypsin inhibitor HC 4	5	4.8	1.07	1.25	1.58	1.49	1.41	NS	0.0010	0.0004	0.0031
LRG1_116844	Leucine-rich alpha-2-glycoprotein 1	4	7.7	1.09	1.73	1.75	1.63	1.54	Not quantitated in naive NHP plasma			
TF_7018	Transferrin	5	2.5	1.02	0.90	0.85	0.86	0.84	NS	0.0035	0.0219	0.0141

*NS* not significant

As seen in Fig. 2a, of the 181 quantified proteins in the EBOV plasma samples, 19 proteins displayed ≥ 2.0 fold expression change in 40% of the EBOV cohort. Of those 19 proteins, 8 were not detectable in naïve NHP plasma (see Table 1a). For the *Bp*-infected NHPs, a total of 158 proteins were quantitated and of these, 14 proteins exhibited ≥ 2 fold abundance change in 40% of the cohort (2/5 NHPs). Among those 14 proteins, 6 were not detectable in naïve plasma sets (see Table 1b). Finally, a total of 154 proteins were quantitated in the naïve cohort and none displayed abundance ratios ≥ 2 fold during the 9-day sampling period.

As shown in Fig. 2a a total of 41 proteins displayed significant alterations in abundance during EBOV infection when compared to naïve NHPs. This list of 41 proteins includes the 11 with abundance changes ≥ 2 fold shown in Table 1a and 30 additional proteins shown in Table 2a. For the *Bp* cohort, a total of 28 proteins displayed significantly changed expression levels when compared to naïve NHPs (see Tables 1b and 2b). Finally, protein levels for the EBOV- and *Bp*-infected plasma sets were compared for all proteins that exhibited significantly changed abundances on overlapping sample collection days (Day 3, Day 5) and Day 6/7 PI. Proteins which exhibited significantly different levels during EBOV- versus *Bp*-infection are shown in Table 3a, b.

**Table 3:** Plasma proteins with significant abundance differences during EBOV versus *Bp* infection (a) in NHP (b) that are attributed to a temporal differences in the host response

Protein description	*p* Value EBOV versus Bp			Trend
	Day 3	Day 5	Day 6/7	
Actin, cytoplasmic 2	NS	NS	0.0129	Higher in EBOV
Apo B48	0.0129	0.0017	0.0004	Higher in EBOV
Apo E	NS	NS	0.0008	Higher in EBOV
Ceruloplasmin	0.0057	0.0128	NS	Higher in Bp
Clusterin	NS	NS	0.0004	Higher in EBOV
Comp factor B	NS	NS	0.0130	Higher in EBOV
Complement C1r	NS	0.1501	0.0173	Higher in EBOV
Complement C2	NS	NS	0.0111	Higher in EBOV
Complement C3	NS	0.0543	0.0292	Higher in Bp
Complement C4A	0.0137	0.0004	0.0004	Higher in Bp
Complement C4B	0.0103	0.0028	0.0004	Higher in Bp
Complement C5	NS	0.0015	0.0101	Higher in Bp
Fibrinogen alpha	0.0010	0.0037	0.0004	Higher in Bp
Fibrinogen beta	0.0004	0.0010	0.0004	Higher in Bp
Fibrinogen gamma	0.0014	0.0026	0.0004	Higher in Bp
Galectin-3 binding protein	NS	NS	0.0200	Higher in EBOV
Leucine-rich alpha-2 glycoprotein 1	NS	0.0057	0.0014	Higher in EBOV
S100 calcium binding protein A9	NS	NS	0.0253	Higher in EBOV

Protein description	*p* Value EBOV versus Bp		
	Day 3	Day 5	Day 6/7
A1AT member 1	0.0015	0.0142	0.1004
A1AT member 3	0.0156	NS	0.0328
Afamin	0.0137	NS	NS
Albumin	0.0066	NS	NS
Apo H	0.0004	NS	NS
Complement C9	0.0230	NS	NS
C-reactive protein	0.0253	NS	NS
Fibronectin-1	0.0004	NS	NS
Hemopexin	NS	NS	0.0161
Inter-alpha-trypsin inhibitor heavy chain 1	0.0027	NS	NS
Inter-alpha-trypsin inhibitor heavy chain 3	0.0419	0.0230	NS
Inter-alpha-trypsin inhibitor heavy chain 4	NS	0.0111	0.0556
Plasminogen	NS	NS	0.0014
Serum amyloid A2	0.0004	NS	NS

### Acute phase plasma proteins with comparable abundance changes during EBOV or *Bp* infection

**Fig. 3:**
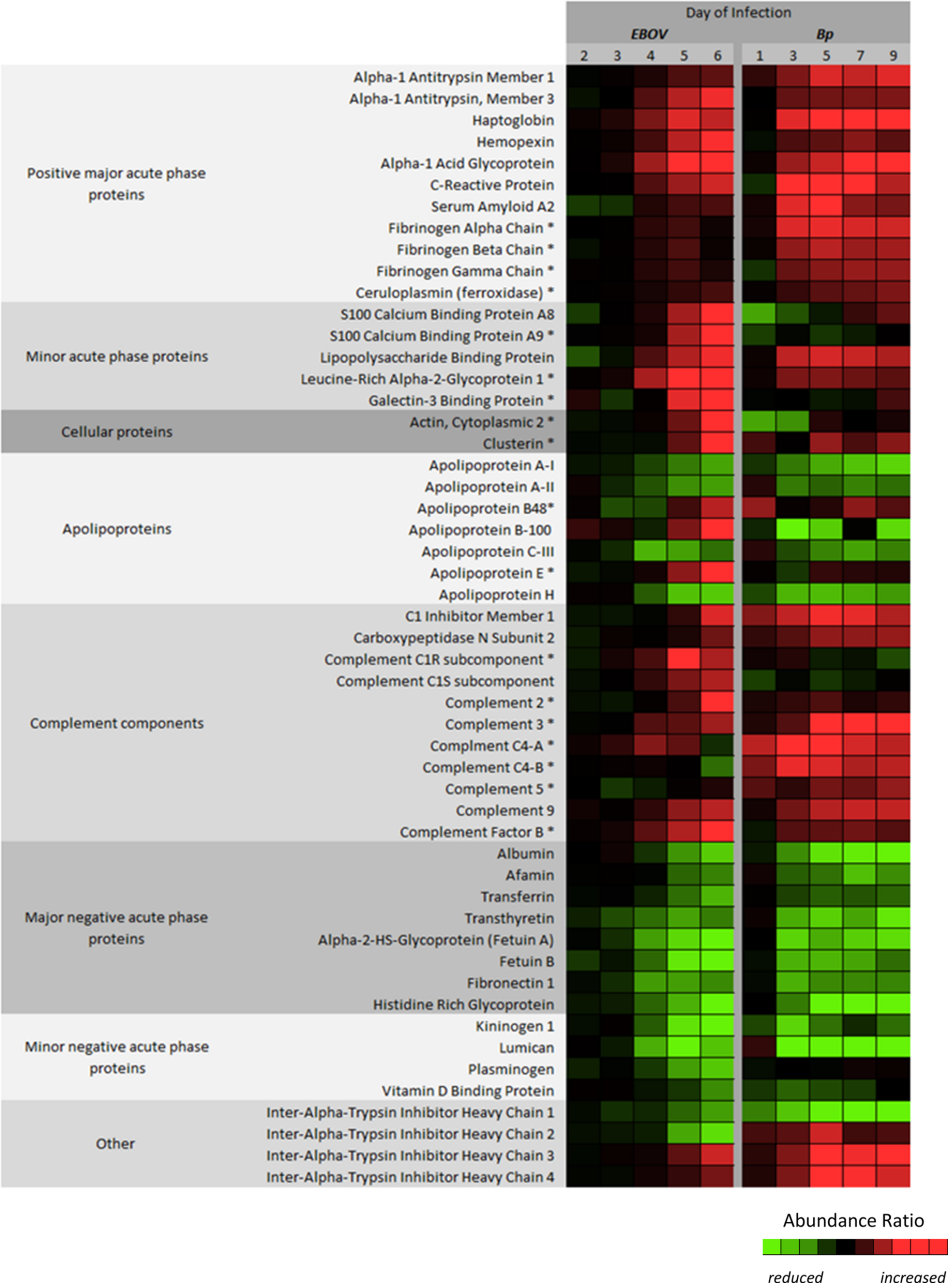
Heat map representing the protein abundance differences observed between EBOV and *Bp* infection. The protein abundance ratio values taken from Tables 1a/b and 2a/b were individually scaled to aid in the visualization of the protein abundance differences observed between EBOV and *Bp* infected plasma. This was done by assigning the lowest ratio values between the 2 sets to light green and the highest to bright red. As a result the heat map illustrates the general trending expression of each class of proteins. Proteins designated with an asterisk (*) had levels that were significantly different based on a 2-way ANOVA test between the two cohorts for at least one common post-infection time-point

For visualization of the datasets, a heat map was constructed using the average fold change values for all proteins which exhibited significant alteration during EBOV or *Bp* infection (see Fig. 3). The proteins are divided up into categories to aid in the reporting of the results and facilitate the comparison of the NHP plasma proteomic response to EBOV or *Bp* infection. The fold change values for each protein represented in the heat map can be found in Tables 1a/b and 2a/b. In general many acute phase plasma proteins were altered to comparable levels during EBOV or *Bp* infection in NHP. However, we observed differences in the temporal kinetics of the proteomic response, in that for most of the quantitated proteins, the *Bp*-infected animals displayed abundance changes 1 day earlier than the EBOV-infected NHP (i.e. Day 3 PI vs. Day 4 PI in the EBOV NHP).

Acute phase proteins, such as C-reactive protein (CRP), Serum Amyloid A2 (SAA2), and Lipopolysaccharide binding protein (LPS-BP) were increased several fold in both infection types, but were not present at high enough levels to be quantitated in the naive NHP plasma samples (see Fig. 2 and Table 1a, b). The major acute phase proteins Alpha-1 antitrypsin (A1AT) members 1 and 3, Haptoglobin, Hemopexin, Alpha-1 acid glycoprotein, CRP, and SAA2 reached comparable maximum fold change values at the common post-infection time-points between the infection types. However, fold change values at the early (Day 3) or late (Day 6/7) PI time-points were often statistically significant between the two cohorts. For example, as shown in Fig. 2b, levels of A1AT3 in the *Bp* cohort were significantly higher than levels found in the EBOV cohort on Day 3 PI, but both infection types reach similar abundance levels on Day 4/5 PI (see Fig. 3 and Table 1a, b). A1AT3 levels continued to increase in the EBOV cohort through Day 6 PI but remained relatively static in the *Bp* cohort. The levels of most positive acute phase proteins in *Bp*-infected NHP reached a maximum between Days 3–5 PI, at which point they either plateaued or exhibited decreased abundance. This may indicate a resolution of the acute response to *Bp* infection, since 4/5 *Bp*-infected animals survived to the end-point of the study (Day 46 PI). As seen in Fig. 2c, d, CRP and SAA2 levels rose dramatically in *Bp*-infected NHP, but a decline is observed afterward over the course of the sampling period. In contrast, in EBOV-infected NHP, levels of SAA and CRP began to increase on Day 3 or 4 PI, and reached maximum levels on Day 5 or 6 PI. Overall a higher amount of variability was observed in the protein abundance data obtained from the *Bp* cohort, especially for CRP and SAA2, indicating a larger variation in the host response of these animals as compared to the EBOV-infected cohort. This is in agreement with previous studies of melioidosis in rhesus macaques and other NHP species which have reported moderate-to-severe, variable disease presentation [24, 26, 27].

In summary, the levels of the major acute phase reactant proteins A1AT1, A1AT3, haptoglobin, hemopexin, A1AGP, CRP and SAA2 were increased in response to EBOV or *Bp* infection. In EBOV-infected NHPs, there was a slightly delayed time dependent increase for these acute phase reactant proteins, but the change in abundance was comparable to *Bp* infected animals. All plasma proteins with abundance differences between the two infection types attributed to temporal kinetics are shown in Table 3b.

### Acute phase plasma proteins with differing abundance levels during EBOV or *Bp* infection

**Fig. 4:**
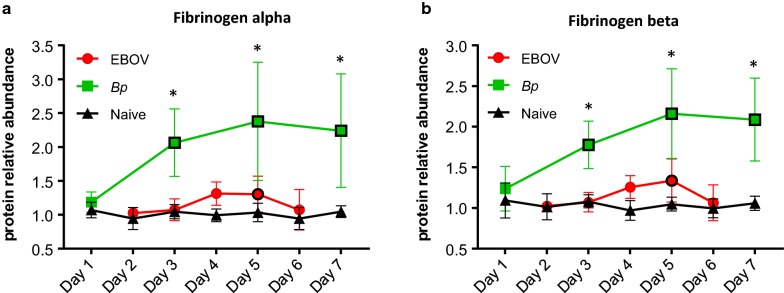
Higher plasma fibrinogen levels were observed during *Bp* infection when compared to EBOV infection in macaques. Levels of **a** fibrinogen alpha and **b** fibrinogen beta were significantly lower in plasma samples collected from rhesus macaques during EBOV infection when compared to *Bp*-infected animals. Abundance levels that were significantly different from levels found in naïve NHPs are designated with a black border around the symbol and levels that were significantly different between EBOV- and *Bp*-infected NHPs are designated with an asterisk (*). Statistical significance was based on 2-way ANOVA analysis

Fibrinogen is a soluble plasma protein synthesized in hepatocytes. Three separate genes encode three distinct polypeptide chains (α, β and γ) forming a homodimer which circulates in the blood and aids in clot formation [31]. In diseases associated with vascular disruption, infection or inflammation, the blood concentration of fibrinogen increases several fold and is considered an acute phase protein [45]. Furthermore, an expanding body of evidence suggests that fibrinogen acts as a mediator of inflammation by interacting with different cell types through cell-specific receptors to induce specific inflammatory functions [32]. An increase in Fibrinogen α, β, and γ chains was observed in both EBOV- and *Bp*-infected NHP plasma. However, the levels of all three Fibrinogen polypeptides were significantly higher in *Bp*-infected NHPs (see Figs. 3 and 4). As seen in Table 1b, and Fig. 4a, b, on Day 3 PI Fibrinogen α and β were increased 2.3 and 1.9 fold respectively in *Bp*-infected NHPs, and Fibrinogen γ abundance levels (see Table 2b) were increased 1.6 fold. Although Fibrinogen α, β, and γ levels were also increased in EBOV-infected NHPs (see Table 2a), the maximum fold change increase was only ≈ 1.4 on Day 5 PI. Not surprisingly, Fibrinogen levels in the EBOV-infected animals returned to baseline levels on Day 6 PI, which is likely a result of Fibrinogen consumption as EVD progresses. As shown in Table 3a, the increased abundance observed for Fibrinogen α, β, and γ in the *Bp* cohort was significantly higher than levels found in the EBOV NHP cohort for all of the overlapping time points (Days 3, 5 and 6/7 PI).

Levels of Ceruloplasmin (CP) or ferroxidase were also higher in *Bp*-infected animals. CP is an acute phase reactant that increases in concentration in serum/plasma during infection and inflammation [27, 28]. As shown in Table 2a, CP abundance increased to significant levels in the EBOV-infected NHPs on Days 5 and 6 PI. *Bp*-infected NHPs displayed a significant increase in abundance on Days 3 and 5 PI and the level of CP increased to a maximum of 1.7 fold on Day 9 PI (see Table 2b). CP levels were significantly higher in the *Bp* cohort on Day 3 PI (*p* = 0.0057) and Day 5 PI (*p* = 0.0128) when compared to EBOV-infected NHPs (see Table 3a and Additional file 1: Figure S1).

The calcium binding proteins S100A8 and S100A9 are secreted into circulation by neutrophils and form a heterocomplex (calprotectin) which is involved in a wide range of cellular processes of innate immunity against microbial invaders [33]. During infection and/or tissue injury their levels are markedly increased [46, 47]. In EBOV-infected NHPs, S100A9 was increased 8.43 fold from pre-infection levels by Day 6 PI, and S100A8 levels increased by 4.95 fold (Table 1a). A comparison with the naïve cohort could not be performed since neither protein was detected in plasma from uninfected animals. In the *Bp*-infected cohort, S100A9 was quantitated in only 2/5 animals and a maximum fold change of 5.85 was observed on Day 9 PI (Table 1b). As seen in Fig. 3, and Additional file 1: Figure S1, in comparison with *Bp*-infected NHPs, S100A9 levels were significantly higher in EBOV-infected animals on Day 6 PI (Table 3a, *p* = 0.0253). Unfortunately, S100A8 was quantitated in only 1 *Bp*-infected NHP, and a maximum fold increase of 1.57 was observed on Day 9 PI for this animal. Therefore, the levels of both S100A8 and S100A9 were higher in EBOV-infected NHP at Day 6/7 PI in comparison to *Bp*-infected animals.

**Fig. 5:**
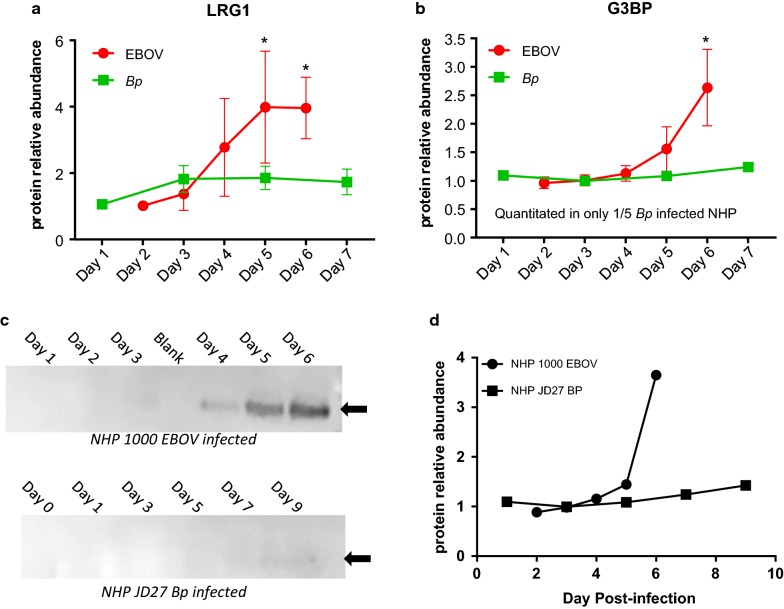
Leucine-rich alpha-2 glycoprotein 1 (LRG1) and galectin-3 binding protein (G3BP) increase during EBOV infection in rhesus macaques. Neither protein was quantitated in naïve NHP plasma **a** Leucine-rich alpha-2 glycoprotein (LRG1) abundance levels increased to > 3 fold on Day 5 PI during EBOV infection whereas, *Bp*-infected NHPs exhibited a very slight increase. LRG1 levels were significantly higher in EBOV-infected animals on Days 5 and 6/7 PI (indicated with * symbol, based on 2-way ANOVA analysis). **b** G3BP abundance increased in EBOV-infected NHPs by > 2.5 fold on Day 6 PI, and was only quantitated in 1/5 *Bp*-infected animals. **c** Representative data from EBOV-infected NHP 1000 depicting Western blot confirmation of the FASP/TMT increased abundance of G3BP observed during EBOV infection. Equal volumes of plasma (5 μl) were loaded for each sample. No bands are visible in plasma from *Bp*-infected NHP JD27, confirming baseline levels and no increase during *Bp* infection. **d** FASP/TMT quantitation for G3BP for NHP 1000 (EBOV-infected: filled circle) and NHP JD27 (*Bp*-infected: filled square)

Leucine-Rich Alpha-2-Glycoprotein 1 (LRG1) is expressed during neutrophilic granulocyte differentiation and is involved in protein–protein interactions, signal transduction, and cell adhesion [34]. LRG1 was not quantitated in the naïve cohort, but in EBOV-infected animals a 3.9-fold increase on Day 5 PI was observed (see Fig. 5a and Table 1a). In the *Bp* cohort, LRG1 was increased 1.7 fold on Day 3 PI (see Table 2b), and when comparing LRG1 levels in EBOV- and *Bp*-infected NHPs, significant *p*-values were obtained for Days 5 and 6 PI (see Table 3a and Fig. 5a), confirming a higher level in EBOV-infected animals for LRG1 at these time-points.

Plasma levels of Galectin-3 binding protein (G3BP), also known as Lectin galactoside-binding soluble-3 binding protein, were significantly higher in EBOV-infected rhesus macaques when compared to *Bp*-infected animals. G3BP was detected and quantitated in 7/10 EBOV-infected NHPs but was quantitated in only 1 *Bp*-infected animal, and was not quantitated in any plasma sample from the naïve cohort. In EBOV-infected animals G3BP increased 2.6 fold by Day 6 PI (see Table 1a and Fig. 5b). Although the ANOVA comparison between the 7 EBOV- and 1 *Bp*-infected NHPs indicated that there was a significantly higher abundance of G3BP on Day 6 PI in EBOV infected NHP (see Table 3a), due to a lack of G3BP TMT quantitation in 4/5 *Bp*-infected plasma sets, an orthogonal confirmation was undertaken. Western blot analysis was performed on 5 EBOV plasma sets and 3 *Bp* plasma sets using a G3BP specific monoclonal antibody. A time-dependent increase in G3BP protein abundance on Days 4-6 PI was confirmed in the EBOV-infected plasma (see Fig. 5c). Conversely, G3BP was not detected by Western blot in any of the *Bp* plasma sets. The increase in G3BP expression in EBOV-infected plasma observed via western blot was comparable to the TMT/MS relative abundance shown in Fig. 5d for EBOV-infected NHP 1000, whereas in *Bp*-infected NHP JD27, G3BP levels remained close to pre-infection levels throughout sampling period. All 5 EBOV-infected plasma sets tested for G3BP via Western blot, exhibited the same trend of increased abundance.

In summary, Fibrinogen and CP levels were significantly higher in *Bp*-infected NHP when compared to EBOV-infected animals. Additionally, there is a time dependent increase in the expression of the plasma proteins S100A8, S100A9, LRG1 and G3BP in EBOV-infected NHP while the *Bp* infected cohort showed no significant induction of these proteins, suggesting a pathogen specific host response.

### Cellular proteins

Some cellular/non-secreted proteins exhibited significant differences in abundance during the late phase of EBOV (Day 6) infection in comparison to *Bp* infection. For example, in EBOV-infected NHPs, cytoplasmic Actin levels reached a 4.4 fold increase on Day 6 PI (see Table 1a). Conversely, Actin levels in *Bp*-infected animals reached only 1.2 fold increase from the pre-infection level on Day 5 PI (data not shown). Therefore as seen in Fig. 3 and Table 3a, Actin levels in EBOV-infected NHP plasma were significantly higher than the levels found in *Bp*-infected NHPs on Day 6/7 PI (*p* = 0.0129). This difference most likely reflects a higher level of cell death occurring late in EBOV infection, ultimately leading to an increase in circulating actin levels. Similarly, the abundance level of Clusterin in EBOV-infected NHPs was significantly elevated on Days 5 and 6 PI and when compared to levels in *Bp*-infected NHP, were higher on Day 6/7 PI (*p *= 0.0004, see Table 3a). Clusterin is associated with the clearance of cellular debris and apoptosis; as such, it is not surprising that increased levels of this protein were detected in plasma from NHPs in the mid-to late stages of EVD.

### Apolipoproteins

**Fig. 6:**
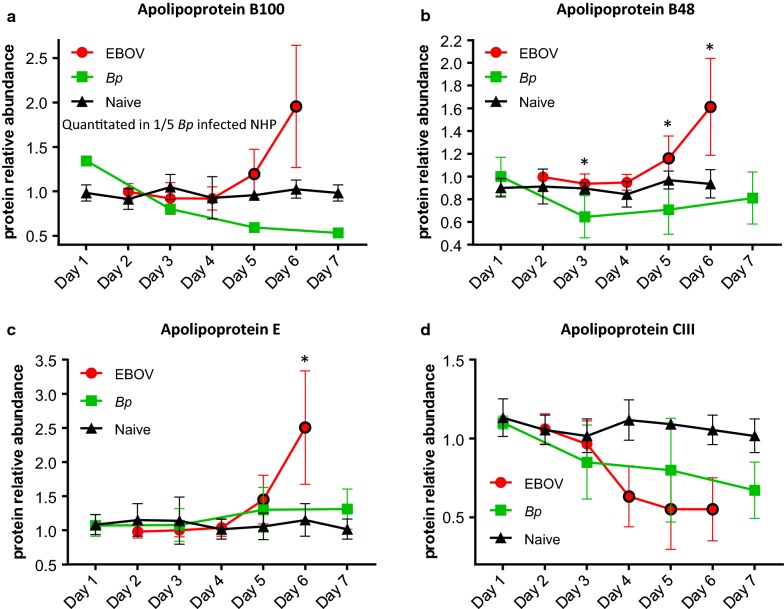
Altered apolipoprotein levels during EBOV or *Bp* infection in rhesus macaques. Abundance levels of Apo B100 (**a**), Apo B48 (**b**) and Apo E (**c**) were increased in EBOV-infected NHPs on Days 5 and 6 PI. No increase was observed for these proteins in *Bp*-infected NHPs. **d** Apo CIII abundance was reduced significantly during EBOV infection, whereas only a slight decrease in abundance was observed in *Bp*-infected NHPs. Abundance levels that were significantly different from levels found in naïve NHPs are designated with a black border around the symbol and levels that were significantly different between EBOV- and *Bp*-infected NHPs are designated with an asterisk (*). Statistical significance was based on 2-way ANOVA analysis

Decreased levels of circulating apolipoproteins in human patients with sepsis correlate with the severity of infection [35], indicating that lipoprotein metabolism is strongly influenced by infection, inflammation and sepsis [36]. The Apolipoproteins A-1, A-II, and B are generally considered negative acute phase reactants [37, 38]. During EVD in rhesus macaques, we observed decreased levels of Apo A1 (see Additional file 1: Figure S2) and Apo AII, however, an increase in the levels of Apo-B100 and Apo-B48 was observed. Due to RNA editing, the Apo B protein occurs in plasma in 2 main isoforms. Apo-B48 and ApoB100 share a common N-terminal sequence, but ApoB48 lacks ApoB100’s C-terminal LDL receptor binding region [39]. In EBOV-infected plasma a significant increase was observed for ApoB100 at Day 5 PI (*p* = 0.0022) and Day 6 PI (*p* = 0.0003) (see Table 1a and Fig. 6a). Unfortunately, Apo B100 was only quantitated in 1/5 *Bp*-infected NHPs and in that animal, a decrease of 1.8 fold was observed on Day 5 PI (see Fig. 6a). Levels of Apo B48 increased on Days 5 and 6 PI in EBOV-infected NHPs (see Table 2a and Fig. 6b), whereas Apo B48 abundance dropped below baseline levels in *Bp*-infected NHPs. This drop in Apo B48 abundance was not significant when compared to the uninfected cohort, but as shown in Table 3a, *Bp*-infected plasma levels of ApoB48 were significantly lower than the EBOV-infected NHPs on Days 3, 5 and 6/7 PI.

Levels of Apolipoprotein E (Apo E) are often elevated in adult and pediatric patients during bacterial infection and sepsis [40, 41]. As shown in Table 1a, abundance levels of Apo E in EBOV-infected NHPs reached a 2.50 fold increase on Day 6 PI. In contrast, as seen in Fig. 6c, the maximum average fold change for Apo E in the *Bp* cohort was 1.24 fold on Day 5 PI, and therefore Apo E levels were significantly higher in EBOV-infected animals on Day 6 PI (*p* = 0.0008, see Table 3a, Figs. 3 and 6c).

Along with Apo A-1 and Apo A-II mentioned above, Apo A-IV, Apo CIII, and Apo H (β2-glycoprotein) abundance levels were significantly reduced in both EBOV-infected and *Bp*-infected NHPs. With the exception of Apo H which exhibited significantly lower levels in the *Bp*-infected NHP on Day 3 PI due to an earlier response, no significant difference was observed for the levels of these Apolipoproteins between EBOV- and *Bp*-infected NHPs at any PI time point. Therefore, in summary, during EVD in rhesus macaques, Apo B100, ApoB48, and Apo E increase in expression and reach higher fold change values than the *Bp*-infected NHP, whereas Apo A1, Apo AII, Apo A4, Apo CIII and Apo H act as negative acute phase reactants in both infection types with comparable reductions.

### Complement cascade components

**Fig. 7:**
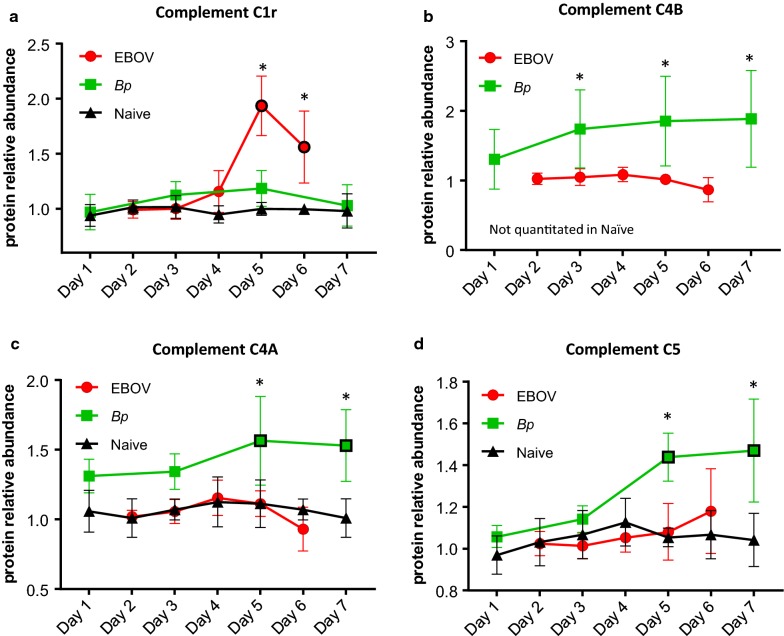
Comparison of complement protein abundance in plasma during EBOV- or *Bp*-infection in rhesus macaques. **a** Abundance levels of C1r were higher in plasma collected from EBOV-infected rhesus macaques on Days 5 and 6 PI. The abundance of **b** C4B, **c** C4A and **d** C5 were significantly higher in plasma samples collected during *Bp* infection when compared to EBOV-infected samples. Abundance levels that were significantly different from levels found in naïve NHPs are designated with a black border around the symbol and levels that were significantly different between EBOV- and *Bp*-infected NHPs are designated with an asterisk (*). Statistical significance was based on 2-way ANOVA analysis

Multiple proteins involved in the complement cascade were identified and quantitated in plasma from EBOV- and *Bp*-infected NHPs, and many abundance differences were observed between the two cohorts. Levels of the complement protein C1r were significantly increased in EBOV-infected NHPs on Days 5 and 6 PI (Table 1a). As seen in Fig. 7a, C1r levels in *Bp*-infected NHPs did increase marginally on Days 3 and 5 PI, but this increase was not significant from levels in naïve animals. The increase of C1r in the EBOV cohort was significantly different from levels in the *Bp* cohort on Days 5 and 6 PI (see Table 3a). Also increased in abundance in the EBOV-infected NHPs were Complement components C1s and C2 on Day 6 PI (Table 2a and Additional file 1: Figure S2B/C). The level of C2 continued to rise in EBOV-infected NHP and was significantly higher on Day 6 PI than levels in the *Bp*-infected animals which remained close to baseline (*p* = 0.0111 see Table 3a).

Major differences in Complement component 4 (C4) levels were observed between EBOV- and *Bp*-infected rhesus macaques. C4 is a polymorphic serum protein consisting of two isoforms, C4A and C4B, which are encoded by two separate genes located within chromosome 6p on the major histocompatibility complex (MHC class III) in both humans and macaques [42, 43]. Complement C4B exhibited the largest abundance increase compared to other complement components in *Bp*-infected NHPs, reaching a 2 fold increase by Day 7 PI (see Table 1b). Unfortunately, this protein was not quantitated in the naïve cohort. In EBOV-infected NHPs, C4B levels were actually reduced 1.3 fold from the pre-infection level (ratio = 0.75) on Day 6 PI. As seen Fig. 7b, although the levels of C4B in the *Bp* cohort had larger variation, the abundance increase was significant from levels observed in the EBOV cohort for Days 3, 5 and 6/7 PI, (*p* = 0.0103, 0.0028 and 0.0004 respectively: see Table 3a). Levels of C4A were also higher in the *Bp*-infected cohort. As seen in Table 2b and Fig. 7c, C4A abundance was increased significantly in *Bp*-infected plasma by Day 5 PI, (*p* = 0.0022 see Table 2b). Conversely, C4A levels in the EBOV cohort were not significantly different from the naïve cohort at any time point, and therefore were significantly lower than the C4A abundance observed in the *Bp*-infected cohort, especially for Days 5 and 6/7 PI (*p *= 0.0004, see Fig. 7c and Table 3a).

In addition to C4A and C4B, levels of complement components C5 and C3 were also significantly higher in *Bp*-infected NHPs when compared to EBOV-infected NHPs. As shown in Fig. 7d, on Days 5 and 6/7 PI, C5 levels were significantly higher in *Bp*-infected animals when compared to EBOV-infected NHPs (see also Table 3a, Fig. 3), and when comparing C3 abundance, *Bp*-infected animals displayed significantly higher levels on Day 6/7 PI (see Additional file 1: Figure S2(D)).

Complement proteins Factor B and component C9 were increased in both the *Bp*- and EBOV-infected NHP on Days 3–5 PI. In EBOV-infected NHPs Factor B rose to a significant level on Day 4 PI (*p* = 0.0095, see Table 2a and Additional file 1: Figure S3(A)), and on Day 6 PI, reached a maximum increase of 1.9 fold. In the *Bp*-infected cohort, Factor B was increased modestly from Day 3 to Day 5 PI, and dropped slightly on Day 6 PI (see Table 2b). Due to the continued increase of Factor B observed in the EBOV-infected NHPs, levels of this protein were significantly higher on Day 6 when compared to *Bp*-infected animals (*p* = 0.0130, see Table 3a). Complement C9 abundance rose in both cohorts during infection (see Additional file 1: Figure S3(B)), and reached a similar maximum. Due to the difference in host response kinetics, levels of C9 in the *Bp* cohort were significantly higher when compared to levels observed in the EBOV cohort on Day 3 PI only (*p* = 0.023, see Table 3b).

### Classic negative acute phase plasma proteins

Classic negative acute phase proteins were quantitated in both the EBOV- and *Bp*-infected NHP. For example, Albumin was reduced significantly in EBOV- and *Bp*-infected animals (see Table 2a, b), and was significantly lower in the *Bp* cohort on Day 3 PI (*p* = 0.0066, see Table 3b), but the EBOV cohort reached a comparable reduction on Days 5-7 PI. Similarly, levels of Transferrin, Afamin, Fetuin A (Alpha-2-HS Glycoprotein), Fetuin B and Transthyretin were reduced in both infection types, and no statistical difference was found between their levels. Levels of Fibronectin 1 were significantly reduced in both infection types, and reached comparable reductions, but due to an earlier drop in levels in the *Bp*-infected animals, levels of Fibronectin 1 were significantly lower when compared to the EBOV-infected NHP on Day 3 PI (*p* = 0.0004 see Table 3b and Additional file 1: Figure S3C).

The inter-alpha (globulin) inhibitor (ITI) family (also known as the family of inter-alpha-trypsin inhibitors) is composed of serine protease inhibitor proteins that are assembled from two precursors: a light chain and either one or two heavy chains [44, 45]. During infection and inflammation, ITI family members can act as both positive and negative acute phase reactants under various disease conditions including sepsis and cancer [46, 47]. We detected and quantitated inter-alpha (globulin) inhibitor H1, H2, H3 and H4 during EBOV and *Bp* infection in rhesus macaques. In this study, ITIH1, ITIH2, were found to be negative acute phase reactants while ITIH3, and ITIH4 acted as positive acute phase proteins during both infection types in NHPs (see Fig. 3, Tables 1b and 2a).

In summary for most negative acute phase proteins, comparable reductions were observed in both infection types. This includes, Albumin, Fibronectin 1, the Fetuins A/B, and the inter-alpha-trypsin inhibitors ITIH1 and ITIH2.

## Discussion

Our approach to the characterization of the rhesus macaque plasma host response to infection is novel in comparison with other studies because we have 1) sampled naïve NHP to better define the variability of acute phase proteins during the sampling period, and 2) compared the rhesus proteomic response in two different infection types (EVD vs. melioidosis). The NHP host response to EBOV infection has been reported in previous studies employing transcriptomic approaches, and a few of these studies have detected upregulated gene expression before the appearance of symptoms [9, 48]. However, no comparison was made with other infection types and therefore the specificity of the transcriptomic response is unknown. Comparative analysis from previous published studies indicates that although some proteins involved in the acute phase response (APR) exhibit similar trends of altered expression in multiple infection types, the fold changes exhibited for certain proteins are quite different depending on the pathogenic agent [49]. Our data supports this finding and therefore future investigations hold promise for the identification and development of panels of human APR proteins which may have expression patterns that are unique for infection types or disease states.

The observed differences in the plasma temporal response between EBOV- and *Bp*-infected NHPs is most likely due to the faster replication rate of *Bp* versus EBOV and the different infection routes used for exposure (aerosol vs. IM). These differences resulted in some protein abundance differences between the two infection cohorts at the early (Day 3) or late (Day 6/7) overlapping PI time points. Accordingly, some of the protein differences observed between the two infection types may be attributed to the immune cells which encounter the pathogen first, leading to the activation of different cellular signaling outcomes.

Most of the host proteins quantitated displayed altered abundance levels by Day 4 or 5 PI in EBOV-infected rhesus macaques. This is concurrent with detectable viremia and development of pyrexia in most of the animals. We noted that animals with an early onset of fever (by Day 3 PI) also had altered protein levels beginning on Day 3 PI. This phenomenon is in agreement with a recent transcriptomic study showing cytokine gene expression is concurrent with the onset of fever in EVD [9]. Interestingly, in some NHP, alteration of host response protein levels occurred before pyrexia and detectable viremia. For example, NHP 0469 presented with fever and detectable viremia on Day 4 PI (see Additional file 1: Table S1), but on Day 3 PI the abundance of LRG1 was increased 1.6 fold for this animal. Since our proteomic approach was untargeted, it may be possible to detect altered expression at earlier pre-symptomatic time points using a targeted LC–MS/MS approach.

Our comparison of the plasma host response to EBOV and *Bp* infection has important implications for efforts to discriminate viral from bacterial infections in human samples. Levels of CRP and SAA have been reported to be significantly higher in some bacterial infections when compared to viral infections [50–53]. In this study, CRP and SAA abundance in *Bp*-infected animals was significantly higher than levels found in EBOV-infected NHPs only for Day 3 PI due to a difference in host response kinetics. At the later common time points of Day 5 PI and Day 6/7 PI there was no significant difference in CRP or SAA levels. It can be argued that the pathogenicity of EBOV infection puts EVD in an entirely different class than typical viral infections, since the level of these acute phase proteins has been reported to increase with disease severity [54]. Therefore the observed levels of CRP and SAA during EVD in rhesus macaques may be higher than what would be expected from an infection caused by some common respiratory viruses.

Ceruloplasmin (CP) levels were significantly higher in *Bp*-infected NHP on Day 3 and 5 PI when compared to EBOV-infected animals. CP is a multi-copper oxidase that is secreted from the liver, involved in iron homeostasis, and accounts for 95% of the copper content of the serum [55]. CP is also an acute phase protein induced in response to inflammation, trauma, or infection with bacteria, viruses and protozoans [56–58]. Since CP is a ferroxidase, it has been proposed that the increased level of this protein during bacterial infections is part of an innate immune strategy to mobilize iron from tissues to starve invading bacterial pathogens of essential iron nutrients [59]. Thus, the observation in our data that plasma CP levels are higher during *Bp* infection versus EBOV infection is in line with this theory and suggests a pathogen specific response.

Both LRG1 and G3BP were increased significantly during EVD in rhesus macaques, when compared to *Bp*-infected NHP. LRG1 is an acute phase protein that is produced by hepatocytes after IL-6 induction and secreted from neutrophils after activation [60]. Levels of LRG1 are increased in many inflammatory conditions including sepsis, appendicitis, rheumatoid arthritis and cancer. Therefore, the higher level of LRG1 may indicate that the degree of inflammation (and neutrophil activation) during infection is greater in EBOV-infected animals than in *Bp*-infected animals. G3BP is a secreted glycoprotein, and although all of its biological functions are not yet fully understood, it has been observed to promote cell-to-cell adhesion, stimulate host-defense against viruses and may be a surrogate biomarker for type 1 interferon-dependent gene activation [61–63]. G3BP is upregulated in many cancers and during infection with several viruses such as HIV, Hepatitis C, Dengue and Hantavirus [64–67]. Our data is in agreement with anti-viral host defense functions, since increased expression was observed during EBOV, but not *Bp* infection. Using ELISA quantitation, Hepojoki et al. found a significant increase in G3BP levels in serum samples from cynomolgus macaques during Hantavirus infection beginning on Day 7 or 8 PI [65]. Additionally, analysis of plasma samples collected from human patients with acute Hantavirus or Dengue infection showed higher G3BP levels when compared to healthy controls [66]. G3BP abundance increases have also been reported during inflammatory conditions such as rheumatoid arthritis and systemic lupus erythematosus, suggesting that chronic bacterial infection could also induce G3BP due prolonged inflammation. Therefore, the examination of plasma at later time points during the chronic phase of *Bp* infection will be necessary to determine if G3BP is increased. Although both LRG1 and G3BP are not filovirus-specific induced proteins, the increased level produced may be useful as blood-based markers to distinguish infection types (bacterial vs. viral) during the acute phase.

Plasma fibrinogen levels in *Bp*-infected NHPs were significantly higher than levels found in EBOV-infected animals. In a transcriptomic analysis reported in 2017 by Liu et al., some of the most highly differentially expressed genes during EBOV infection in humans were fibrinogen alpha, beta, and gamma [68]. Likewise, Ebihara et al. found increased fibrinogen levels in rhesus macaques on Days 4 and 5 of EBOV infection which strikingly declined at the terminal stages of EVD [69]. Plasma fibrinogen levels are known to decline due to the conversion of fibrinogen to fibrin, and EVD causes coagulopathy resembling disseminated intravascular coagulation (DIC) at the terminal stages of disease [70]. However, since fibrinogen is an acute phase reactant produced by the liver, increased levels during inflammation and infection may mask fibrinogen consumption during clot formation. In this study, fibrinogen α, β, and γ were increased during EBOV infection beginning on Day 4 PI and reverted to baseline by Day 6 PI. The increase in fibrinogen protein levels was much higher in *Bp*-infected NHPs beginning on Day 3 PI and remained high through Day 9 PI. Generally, by Day 4 of EVD in rhesus macaques, macrophages, monocytes, dendritic cells, and fibroblasts are infected, but serum chemistry alterations, petechia and uncontrolled hemorrhage usually do not manifest until after Day 4 PI [69, 71]. Additionally, abnormal coagulation measurements (i.e. Prothrombin time: PT and Activated Partial Thromboplastin Time: APTT) have been reported to peak between Days 6 and 10 PI during EBOV infection in rhesus macaques [71]. Collectively, this evidence indicates that the lower fibrinogen level observed during EVD when compared to *Bp* infection is not the result of fibrinogen consumption on Days 4 or 5 PI, while the drop in fibrinogen levels observed on Day 6 PI may be the result of fibrinogen conversion to fibrin. Additionally, although hepatocytes are usually infected by Day 3 or 4 PI during EVD [72], widespread liver damage does not typically appear at this stage of the infection. Therefore fibrinogen abundance observed on Day 4 PI is most likely not affected by a decline in hepatocyte production. Although we observed other acute phase reactants and markers of inflammation to be highly upregulated in EBOV-infected NHPs, the lower level of Fibrinogen in EVD in comparison with *Bp*-infected NHPs is not easily explained. Given the evidence of highly upregulated Fibrinogen transcripts during EBOV infection in humans, it is tempting to speculate that the virus is somehow able to suppress the production of the fibrinogen proteins in circulating plasma.

Our data from NHPs during EVD is in agreement with the lipoprotein alterations observed during sepsis, in that plasma levels of Apo A1 were reduced while Apo E levels increased. In this study true sepsis was not observed in the *Bp*-infected animals, since only 1/5 NHPs had a positive blood culture, and therefore we observed only a slight increase in Apo E abundance. It has been reported that triglyceride levels increase by at least fivefold in EBOV-infected rhesus macaques, but total cholesterol changes are negligible [71]. The increase of Apo B we observed during EBOV infection is likely a direct result of the increase in chylomicron particles carrying plasma triglycerides.

Increases in levels of proteins relevant to all three complement pathways were observed during EBOV and *Bp* infection in rhesus macaques. The ongoing identification of novel mechanisms of viral antagonism of components of the complement system highlights the important role of these pathways in innate immunity. For example, the NS1 glycoprotein of some Flaviviruses (i.e. Dengue, West Nile and Yellow fever), which is expressed on the surface of and secreted by infected cells, can suppress complement by recruiting and activating C1s and C4BP to promote cleavage of C4 and inactivate C4B [73]. Additionally, both soluble and membrane-bound NS1 proteins of Dengue and West Nile viruses have been reported to interact with the complement regulatory component factor H, resulting in decreased deposition of C3 and C5b-9 membrane attack complexes on cell surfaces [74]. Our data indicates that the levels of complement components C1r, C1s, C2, C9 and factor B are significantly increased in plasma during EBOV infection. While there was a slight non-significant increase in the abundance level of C3 and C5. Conversely, *Bp*-infected NHPs displayed increases in C3, C4A, C4B, C5 and C9, which were significantly higher than those found in EBOV-infected NHPs. It is possible that excess complement activation lead to the consumption of C3, C4 (A/B) and C5 in plasma during EVD, or the lower levels observed for these proteins may indicate an antagonism of complement components, specifically those downstream of C1 and C2, by the EBOV virus. An antagonistic strategy that causes the downregulation of the proinflammatory chemoattractants (C3A, C4A and C5A) in plasma during EBOV infection would lead to a reduced influx of inflammatory cells into infected sites, potentially contributing to EVD pathogenesis.

## Conclusions

In conclusion, plasma proteomic analysis uncovered specific differences in acute phase protein levels induced during EVD or melioidosis in rhesus macaques. These differences reflect the global circulating innate immune response to a highly pathogenic viral or bacterial agent in relevant NHP models of infection. Most notable in our results is the higher level of fibrinogen and complement proteins C3, C4, and C5 exhibited by *Bp*-infected NHPs, while EBOV-infected animals displayed higher levels of two glycoproteins: LRG1 and G3BP. Future studies examining the plasma proteomic host response of rhesus macaques to other infection types endemic to the same regions where EBOV and *Bp* are prevalent will facilitate the identification and development of a panel of plasma host proteins that could be used to differentiate infection types. These panels could then be validated using human samples to determine the diagnostic potential.

## Additional file

**Additional file 1.** Supplementary tables and figures.

## Abbreviations

NHP: non-human primate
EVD: Ebola virus disease
EBOV: Ebola virus
*Bp*: *Burkhoderia pseudomallei*
LC–MS/MS: liquid chromatography tandem mass spectrometry
PFU: plaque forming units
CFU: colony forming units
TMT: tandem mass tags
FASP: filter assisted sample prep
HCD: higher energy collisional dissociation
PSM: peptide spectrum match
IM: intramuscular
