# Supplementary material for: Characterization of the plasma proteome of nonhuman primates during Ebola virus disease or melioidosis: a host response comparison
Source: Clin Proteomics. 2019 Feb 7;16:7. doi: 10.1186/s12014-019-9227-3 (PMC6366079; doi:10.1186/s12014-019-9227-3)
Supplement: Supplementary file 1 — Additional file 1. Supplementary tables and figures. [file 12014_2019_9227_MOESM1_ESM.docx]

**Supplementary Materials:**

**Figure S1.** Ceruloplasmin and S100 A9 levels in EBOV- and *Bp* infected NHP

**Fig. S1. Ceruloplasmin and S100A9 levels during infection with EBOV or *Bp*.** A) Ceruloplasmin levels were higher in *Bp* infected NHP when compared to EBOV infected animals on Days 5 and 6 PI. B) S100 A9 levels in EBOV infected NHP were significantly higher than level in Bp-infected animals. Abundance levels that were significantly different from levels found in naïve NHPs are designated with a black border around the symbol and levels that were significantly different between EBOV- and Bp-infected NHPs are designated with an asterisk (*). Statistical significance was based on 2-way ANOVA analysis

**Figure S2.** Apo A1, Complement C1s, C2 and C4 levels.

**Figure S2. Apo A1, Complement C1s, C2 and C4 levels during infection with EBOV or BP in rhesus macaques.**

A) Apo A1 levels decreased to comparable levels in EBOV- and *Bp*- infected NHP. B) Complement C1s levels were higher in EBOV infected NHP, but the difference was not significant. C) Complement C2 levels were significantly higher in EBOV-infected NHP on Day 6 PI. D). Complement C3 levels were significantly higher in *Bp* infected NHP on Day 6/7 PI. Abundance levels that were significantly different from levels found in naïve NHPs are designated with a black border around the symbol and levels that were significantly different between EBOV- and *Bp*-infected NHPs are designated with an asterisk (*).Statistical significance was based on 2-way ANOVA analysis.

**Figure S3.** Complement Factor B, C9 and Fibronectin-1 levels

**Figure S3. Complement factor B, C9 and Fibronectin-1 levels during infection with EBOV or BP in rhesus macaques**

A) Complement Factor B is significantly higher in EBOV infected NHP on Day 6/7 PI. B) Levels of C9 were comparable in both infection types. C) Fibronectin levels are reduced in both EBOV- and *Bp*-infected NHP, and the reduction is significantly different between the two infection types on Day 3 PI. Abundance levels that were significantly different from levels found in naïve NHPs are designated with a black border around the symbol and levels that were significantly different between EBOV- and *Bp*-infected NHPs are designated with an asterisk (*).Statistical significance was based on 2-way ANOVA analysis.

**Table S1.** EBOV viral RNA levels in EBOV infected NHP.

| **Pathogen/exp.route** | **NHP#** | **Copies of serum EBOV RNA ge/ml** | | | | | | |
| --- | --- | --- | --- | --- | --- | --- | --- | --- |
|  |  | **Day 2** | **Day 3** | **Day 4** | **Day5** | **Day 6** | **Day 7** | **Day 9** |
| Ebola Zaire (1000 pfu:IM) | 1509 | <LLOD | 3.44E+06 | 1.45E+08 | 3.22E+08 | ND | 3.76E+08 | ND |
|  | 1500 | <LLOD | 2.47E+05 | 2.42E+07 | 2.44E+09 | ND | 1.54E+09 | ND |
|  | 1285 | <LLOD | <LLOD | 4.13E+06 | 2.63E+08 | ND | 2.45E+08 | 1.18E+08 |
|  | 1315 | <LLOD | 6.41E+05 | 6.41E+08 | 9.29E+09 | ND | 2.06E+10 | ND |
|  | 1305 | <LLOD | <LLOD | 4.30E+06 | 1.12E+09 | ND | 9.94E+09 | 2.83E+09 |
|  | 1129 | <LLOD | <LLOD | 3.51E+05 | 7.79E+08 | ND | 6.70E+09 | 4.16E+09 |
|  | 1120 | <LLOD | 2.10E+05 | 3.24E+08 | 2.78E+10 | 1.60E+11 | ND | ND |
|  | 1456 | <LLOD | 1.87E+06 | 2.19E+09 | 1.58E+10 | 2.28E+10 | ND | ND |
|  | 469 | <LLOD | <LLOD | 2.35E+07 | 4.61E+09 | 6.39E+09 | ND | ND |
|  | 1000 | <LLOD | <LLOD | 1.44E+06 | 2.38E+08 | ND | 5.68E+09 | 2.25E+09 |

ND = No Data (sample not collected)

**Table S2.** Blood culture results for *Bp* infected NHP.

| **Pathogen/exp.route** | **NHP#** | **Blood culture results (CFU/ml)** | | | | | | | | |
| --- | --- | --- | --- | --- | --- | --- | --- | --- | --- | --- |
|  |  | **Day 0** | **Day 2** | **Day 3** | **Day 4** | **Day5** | **Day 6** | **Day 7** | **Day 8** | **Day9** |
| Burkholderia (300-500 cfu: Aerosol) | JD 27 | 0.0 | 0.0 | 0.0 | 0.0 | 0.0 | 2.5 | 0.0 | 0.0 | 0.0 |
|  | JF18 | 0.0 | 0.0 | 0.0 | 0.0 | 0.0 | 0.0 | 0.0 | 0.0 | 0.0 |
|  | JH28 | 0.0 | 0.0 | 3.3 | 0.0 | 0.0 | 0.0 | 0.0 | 0.0 | 0.0 |
|  | JM 26 | 0.0 | 0.0 | 0.0 | 0.0 | 0.0 | 0.0 | 0.0 | 0.0 | 0.0 |
|  | JP28 | 0.0 | 0.0 | 0.0 | 0.0 | 0.0 | 0.0 | 0.0 | 0.0 | 0.0 |

**Table S3.** Bacterial burden in terminal tissues and urine from *Bp* infected NHP.

| **Pathogen/exp. Route** | **NHP #** | **Day** | **Terminal Tissue (CFU/g) and urine (CFU/ml) burden** | | | | | | |
| --- | --- | --- | --- | --- | --- | --- | --- | --- | --- |
|  |  |  | Lung | Bronch LN | Spleen | Liver | Mes LN | Kidney | Urine* |
| Burkholderia (300-500 cfu: Aerosol) | JD 27 | 13 | 1.60E+07 | 2.90E+04 | 3.70E+04 | 1.40E+04 | 5.60E+01 | 2.90E+02 | 0 |
|  | JF18 | 46 | 0 | 8.20E+04 | 0 | 0 | 0 | 0 | 0 |
|  | JH28 | 46 | 0 | 5.10E+04 | 2.60E+03 | 0 | 0 | 0 | 0 |
|  | JM 26 | 46 | 0 | 1.50E+02 | 5.80E+03 | 0 | 5.60E+01 | 0 | ND |
|  | JP28 | 46 | 4.00E+02 | 2.60E+04 | 4.30E+05 | 2.00E+05 | 8.00E+02 | 4.70E+03 | 4.00E+01 |
